# Harnessing Non-Antibiotic Strategies to Counter Multidrug-Resistant Clinical Pathogens with Special Reference to Antimicrobial Peptides and Their Coatings

**DOI:** 10.3390/antibiotics14010057

**Published:** 2025-01-09

**Authors:** Shyam Kumar Mishra, Tanzina Akter, Umme Laila Urmi, George Enninful, Manjulatha Sara, Jiawei Shen, Dittu Suresh, Liangjun Zheng, Elias Shiferaw Mekonen, Binod Rayamajhee, Francesco M. Labricciosa, Massimo Sartelli, Mark Willcox

**Affiliations:** 1 School of Optometry and Vision Science, Faculty of Health and Medicine, University of New South Wales, Sydney, NSW 2052, Australia; s.mishrabaishnab@unsw.edu.au (S.K.M.); t.akter@unsw.edu.au (T.A.); manjulatha.sara@unsw.edu.au (M.S.); jiawei.shen@unsw.edu.au (J.S.); b.rayamajhee@unsw.edu.au (B.R.); m.willcox@unsw.edu.au (M.W.); 2 Department of Microbiology, Tribhuvan University Teaching Hospital, Institute of Medicine, Kathmandu 44600, Nepal; 3 Microbial Biotechnology Division, National Institute of Biotechnology, Dhaka 1349, Bangladesh; 4 School of Chemistry, Faculty of Science, University of New South Wales, Sydney, NSW 2052, Australia; 5 Department of Animal Science and Technology, University of Northwest A&F, Yangling 712100, China; 6 Global Alliance for Infections in Surgery, 62100 Macerata, Italy; 7 Department of Surgery, Macerata Hospital, 62100 Macerata, Italy

**Keywords:** antimicrobial coating, antimicrobial peptide, antimicrobial resistance, peptide engineering, peptidomimetics

## Abstract

Antimicrobial resistance is a critical global challenge in the 21st century, validating Sir Alexander Fleming’s warning about the misuse of antibiotics leading to resistant microbes. With a dwindling arsenal of effective antibiotics, it is imperative to concentrate on alternative antimicrobial strategies. Previous studies have not comprehensively discussed the advantages and limitations of various strategies, including bacteriophage therapy, probiotics, immunotherapies, photodynamic therapy, essential oils, nanoparticles and antimicrobial peptides (AMPs) within a single review. This review addresses that gap by providing an overview of these various non-antibiotic antimicrobial strategies, highlighting their pros and cons, with a particular emphasis on antimicrobial peptides (AMPs). We explore the mechanism of action of AMPs against bacteria, viruses, fungi and parasites. While these peptides hold significant promise, their application in mainstream drug development is hindered by challenges such as low bioavailability and potential toxicity. However, advancements in peptide engineering and chemical modifications offer solutions to enhance their clinical utility. Additionally, this review presents updates on strategies aimed at improving the cost, stability and selective toxicity of AMPs through the development of peptidomimetics. These molecules have demonstrated effective activity against a broad range of pathogens, making them valuable candidates for integration into surface coatings to prevent device-associated infections. Furthermore, we discuss various approaches for attaching and functionalising these peptides on surfaces. Finally, we recommend comprehensive in vivo studies to evaluate the efficacy of AMPs and their mimetics, investigate their synergistic combinations with other molecules and assess their potential as coatings for medical devices.

## 1. Introduction

Globally, multidrug-resistant (MDR) pathogens have emerged as one of the most critical threats to public health with approximately 5 million deaths associated with bacterial antimicrobial resistance in 2019 [1]. According to the World Health Organization (WHO), antimicrobial resistance occurs when microbes including bacteria, viruses, fungi or parasites do not respond to antimicrobial agents that are commonly prescribed to kill them or prevent their growth [2]. Drug (antimicrobial) resistance is a growing concern in nearly all pathogenic microbes (bacteria, viruses, fungi and protozoa) [3]. In particular, bacteria are called MDR when they show resistance to one or more antimicrobial agent(s) from three or more classes of antimicrobial drugs [4]. Among the MDR bacteria, ESKAPE pathogens (*Enterococcus faecium*, *Staphylococcus aureus*, *Klebsiella pneumoniae*, *Acinetobacter baumannii*, *Pseudomonas aeruginosa* and *Enterobacter* spp.) are major concerns as they cause mild to severe life-threatening infections and often lead to prolonged illness, increased mortality and high economic burden in healthcare settings worldwide [5,6].

## 2. Antimicrobial Resistance in Bacteria

Bacterial cells employ various mechanisms to inactivate antibiotics, including decreased influx, increased efflux, the modification of antibiotics and the prevention of antibiotics from binding to their targets [7]. Alterations in membrane structures and membrane proteins can influence cell membrane permeability, thereby impacting the influx and efflux of antibiotics. For instance, reduced expression of the *ompF* gene leads to reduced OmpF porin in the cell membrane, which decreases its permeability in *Salmonella* Muenchen, leading to resistance against β-lactam antibiotics [8]. β-lactamases are a representative example of resistance mechanisms that modify antibiotic functional groups, as these enzymes confer resistance to β-lactam drugs by hydrolysing the amide bond of the β-lactam ring, thereby degrading the drug [9]. Additionally, the modification of antibiotics can prevent binding to their targets, as seen with aminoglycosides by bacterial aminoglycoside-modifying enzymes, which significantly reduce the drug’s affinity for its target [10]. Moreover, blocking the binding pathway of antibiotics to their targets through modification or protection of the targets is another resistance mechanism. An example of this is the methylation of 16S rRNA, which confers high-level resistance to aminoglycoside antibiotics [11]. Antimicrobial resistance mechanisms in bacteria can be categorised on phenotypic and genetic bases.

### 2.1. Phenotypic Resistance to Antimicrobials

Phenotypic resistance refers to changes in bacterial behaviour and physiological state that allow them to exhibit temporary resistance, tolerance or persistence when exposed to antibiotics, without permanent alteration of their genetic materials. One of the most common phenotypic adaptations is biofilm formation. Biofilms are microbial populations adhered to surfaces that provide a physical barrier, where cells in biofilms show greater resistance/tolerance to antimicrobial agents compared to planktonic (free living) cells [12]. Patients infected with strong biofilm producers may have longer hospital stays than those infected with weak or non-biofilm producers [13]. In addition to biofilm formation, bacterial populations can withstand transient exposures to high doses of bactericidal antibiotics without a change in the minimum inhibitory concentration (MIC) by becoming tolerant. Tolerant bacteria may alter their metabolic activity entering a dormant state that renders them less susceptible to antibiotics that typically target actively growing cells. Upon removal of the antibiotic exposure, they return to their previous state [14,15]. Another physiological adaptation is ‘persister’ bacteria, which do not respond to antibiotics and are not killed, although they are unable to multiply in the presence of bactericidal antimicrobials. Upon treatment cessation, these persistent subpopulations resume their growth, leading to relapsing or chronic infection [15,16,17]. Similarly, outer membrane vesicle formation around Gram-negative bacterial cells can sequester cationic AMPs and antibiotics, thereby inhibiting their activity against the bacterial cell and leading to transient resistance [18].

### 2.2. Genetic Basis of Resistance to Antimicrobials

Genetic resistance includes both intrinsic and acquired mechanisms [19].

#### 2.2.1. Intrinsic Resistance

Intrinsic resistance is a natural bacterial trait resulting from their normal genetic, anatomic or physiologic state. It does not rely on previous exposure to antibiotics, nor does it result from the acquisition of traits through horizontal gene transfer. Examples include vancomycin resistance in *E. coli* (where the outer membrane provides a barrier to the drug’s entry) or aminoglycoside resistance in anaerobes [20,21,22]. Such resistances may be associated with the absence of a receptor for the antibiotic, reduced affinity, cell wall impermeability or enzyme production [23].

#### 2.2.2. Acquired Resistance

Antibiotic resistance that arises from alterations in the physiology and anatomy of bacterial cells, driven by changes in genetic makeup, is classified as acquired resistance. Since this resistance is unpredictable in organisms, clinical laboratories determine the antibiogram of such isolates. While intrinsic resistance mechanisms are encoded in the bacterial chromosome, acquired resistance is typically gained through horizontal gene transfer (HGT) via transformation (incorporation of naked DNA), transduction (phage-mediated transfer) or conjugation (sex-pili-mediated genetic exchange, e.g., of plasmids) [19,24]. Among these mechanisms, transformation is the simplest form of HGT, although it is exhibited by a limited number of bacterial species. Conjugation is highly efficient and can contribute to the emergence of resistance in hospitals. Plasmids and integrons, as key mobile genetic elements, play crucial roles in the dissemination of antimicrobial resistance among clinically important bacteria [25]. Additionally, resistance can emerge through successful genetic mutations in gene(s) associated with the antimicrobial molecule’s activity [25].

### 2.3. The Burden of Antimicrobial Resistance

**Figure 1:**
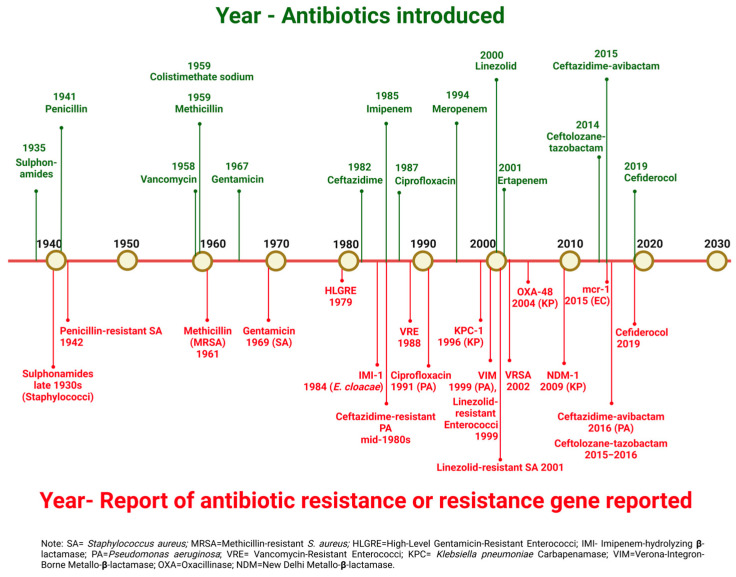
Timeline of introduction of antibiotics and resistance development. (Created in https://BioRender.com, accessed on 25 December 2024).

According to a recent systematic review and meta-analysis on the burden of antibiotic resistance, the attributable cost per patient episode for an antibiotic-resistant infection ranges from US$ 2371.4 to + US$ 29,289.1 (adjusted for 2020 prices). The mean excess length of hospital stay due to these infections is 7.4 days (95% CI: 3.4–11.4). Furthermore, the odds ratio for mortality associated with resistant infections is 1.844 (95% CI: 1.187–2.865) [26]. On one hand, there is a high burden of antimicrobial-resistant infections; on the other hand, there is a paucity of new antibiotics in the pipeline. Figure 1 illustrates the year of introduction of different antibiotics into clinical practice and the subsequent years when antibiotic resistance or corresponding resistance genes were first reported.

In many countries with a high prevalence of MDR infections, access to both newer and effective older antibiotics is inadequate [27]. To mitigate the threat of antimicrobial resistance, infection control and prevention, along with antimicrobial stewardship strategies, are essential. However, discovering novel antimicrobial agents is also crucial to effectively address this challenge [28].

## 3. Alternative Non-Antibiotic Approaches for the Prevention and Control of MDR Pathogens

Because of emerging antimicrobial resistance, traditional antibiotic therapies are becoming ineffective, therefore alternative non-antibiotic strategies are being explored as potential strategies for managing MDR pathogens.

### 3.1. Bacteriophage Therapy

Bacteriophages are viruses that specifically infect and kill bacteria. They can be utilised to kill infecting MDR pathogens without harming either normal microbiota or eukaryotic host cells. Bacteriophage therapy also results in rapid proliferation within the host bacteria, producing an auto-dosing effect. They can boost the effectiveness of antibiotics, as bacteria respond to the phages by modifying their cell walls and membranes, which can lead to increased susceptibility to antibiotics [29,30]. Unlike antibiotics, one of the advantages of using phages is that they are able to adapt to bacterial resistance development and regain an upper hand over bacteria as they mutate alongside their host [31]. Several in vitro studies have demonstrated the effectiveness of phages as antibacterial agents against biofilm and planktonic bacteria [32,33,34].

Commercial phage preparations, including “Stafal”, “Sextaphage”, “PhagoBioDerm” and “Pyophage” are available to combat ESKAPE pathogens [35]. Stafal, from Bohemia Pharmaceuticals in Slovakia, aims to treat *S. aureus* infections. Russia’s Microgen produces Sextaphage, a cocktail targeting both *P. aeruginosa* and *Escherichia coli* [35]. Pyophage, developed by the Georgian Eliava Institute, aims to fight pathogens that are responsible for causing skin and intestinal infections [35]. PhagoBioDerm is a specialised bandage infused with phages, ciprofloxacin and other active ingredients, designed for the slow-release treatment of wounds and ulcers caused by *S. aureus* and *P. aeruginosa* [36]. Successful phage treatments most commonly occur with tailored bacteriophage therapy [37] as well as combined bacteriophage–antibiotic therapy rather than in randomised controlled trials of non-personalised bacteriophage therapy [29]. For this personalised phage therapy, determination of the phage susceptibility profile (PST) or ‘phagogram’ is required. This requires the development of standard protocols for determining the phagogram, as well as the production of ‘ready-to-use’ stable phage products [38].

### 3.2. Probiotics

Probiotic-based approaches offer a potential strategy to counter MDR bacteria [39,40]. Probiotics are non-toxic viable microorganisms that may be beneficial to the host when consumed in sufficient amounts [41]. The key antimicrobial mechanisms of probiotics are their competitive exclusion of pathogenic bacteria, secretion of antimicrobial molecules against pathogens, immune modulation and improvement of intestinal barrier function by enhancing mucin and tight junction protein expression [42]. Probiotics have been shown to prevent or cure infections of ESKAPE pathogens by competing for colonisation sites [43]. One of the most commonly used probiotics is *Lactipantibacillus plantarum* (*Lactobacillus plantarum*) as it can have growth-inhibiting, bactericidal and anti-biofilm activities [44]. Probiotics have been recognised as a strategy to prevent ventilator-associated pneumonia in critically ill patients [45]. However, emerging safety concerns with the use of probiotics in patient populations warrant meticulous testing to ensure compliance with specific quality benchmarks prior to their use [46].

### 3.3. Immunotherapies

Immunotherapies are designed to boost the host immune system to tackle bacterial infections, offering an alternative approach to traditional antibiotics. This can be achieved with monoclonal antibodies (mAbs), cytokines, immune checkpoint inhibitors and vaccines. Monoclonal antibodies target specific bacterial antigens or virulence factors to improve the immune response [47]. For *P. aeruginosa* and *S. aureus*, numerous tailored mAbs are undergoing clinical trials [48]. For instance, MEDI3902 (AstraZeneca PLC) is a bispecific IgG1 antibody targeting the PcrV protein, which is cytotoxic to host cells, and the Psl exopolysaccharide, which is required for the colonisation and adhesion of *P. aeruginosa* [49]. Another mAb, KB001-A, is a PEGylated mAb fragment specific to the Type III secretion system (TTSS) of *P. aeruginosa* and is used for the treatment of pneumonia in high-risk patients [50]. In cases of *S. aureus* pneumonia, mAb AR-301 (Aridis Pharmaceuticals) neutralises its α-toxin [51]. The development of vaccines and the use of cytokines and immune checkpoint inhibitors against the ESKAPE pathogen have had only modest success because of bacterial strain heterogenicity and different virulence factors employed for pathogenesis, which indicate the necessity of identifying variability in order to obtain significant results [48,52]. In the case of *K. pneumoniae*, a vaccine candidate targeting the outer membrane vesicle has been shown to provide protection in a preclinical animal model [53].

### 3.4. Photodynamic Therapy

Photodynamic therapy is a treatment where a non-toxic photosensitiser and light of a suitable wavelength are used. The photosensitiser is excited with the light and transformed from its ground state of having low energy to an excited triplet state in which the photosensitiser transfers electrons, energy or molecular oxygen to produce reactive oxygen species (ROS) or singlet oxygen radicals. These ROS and oxygen radicals are toxic to bacterial nucleic acid, protein, lipid and polysaccharide components [54,55]. An advantage of using photodynamic therapy is that there is no or very little selective pressure for the development of resistance [56].

Some examples of the most frequently used photosensitisers are phenothiazinium derivatives (methylene blue, toluidine blue and rose bengal), porphyrin, fullerene derivatives and natural photosensitisers (such as hypericin, flavin derivatives and curcumin) [57,58,59]. In vitro research has indicated that blue light exhibits broad-spectrum antibacterial and antibiofilm activity against all ESKAPE pathogens [60]. Repeated exposure to light-emitting diodes reduced *P. aeruginosa* growth [61]. *E. faecium*, when treated with green light and low doses of rose bengal, also exhibited substantial growth reductions [62]. In the case of *A. baumannii*, the natural photosensitisers riboflavin and chlorophyllin under blue light yielded reductions in both planktonic and biofilm cells where riboflavin showed a more potent antibiofilm effect than chlorophyllin due to the production of higher amounts of ROS [63]. The use of riboflavin and ultraviolet light kills several ESKAPE pathogens, with increases in UV fluence producing greater death in vitro [59].

### 3.5. Essential Oils

Plant-derived essential oils (EOs) are volatile, hydrophobic secondary metabolites that can be effective in both planktonic and sessile bacterial forms [64]. Due to their complex structure, containing multiple bioactive compounds with multi-target mechanisms, they exhibit low resistance potential [65]. EOs act by hindering the formation of bacterial biofilms, disrupting quorum sensing (bacterial cross-talk) and blocking efflux pumps [66]. Their lipophilic characteristics enable them to modify bacterial membrane permeability, which may lead to disruption of the membrane [67].

Some common complex oils that have antibacterial activities against different pathogenic bacteria are cassia, clove, eucalyptol, cinnamon, lavender, lemon, orange, oregano, marjoram, peppermint, tea, Peru balsam, rosemary and thyme oil, while individual components include terpinen-4-ol, α-terpineol, eugenol thymol, menthol, pulegone, carvacrol, cinnamaldehyde, citral, citronellol, linalool and linalyl acetate [68]. EOs extracted from *Foeniculum vulgare* and *Ridolfia segetum* can inhibit *P. aeruginosa* biofilms in vitro [69]. Clove oil and peppermint oil showed strong anti-biofilm and anti-virulence properties (inhibition of LasB elastase, protease, chitinase and pyocyanin production) in *P. aeruginosa*, [70] while eugenol was found to affect the production of additional virulence factors such as rhamnolipid and pyoverdine [71]. In addition to *P. aeruginosa*, eugenol has shown anti-biofilm in methicillin-resistant *S. aureus* (MRSA) [72]. EO vapours from *Melaleuca* sp. were found to reduce culturable microbes in interactions with aerosolised *Aspergillus flavus* spores, *E. coli* or surrogates of SARS-CoV-2 and murine hepatitis coronavirus MHV-1, potentially aiding in the control of airborne infections like COVID-19 [73]. Similarly, Eos of ginger, garlic, turmeric and *Ageratina adenophora* (crofton weed) at the concentration of 200 mg/L prevented biofilm formation by MDR clinical *A. baumannii* isolates by 70.8%, 68.6%, 51.9% and 67.6%, respectively [74].

### 3.6. Nanoparticles

Nanoparticles (NPs) have emerged as promising tools for controlling microbial infections because of their unique properties, which include biocompatibility, a high surface-area-to-volume ratio, improved penetration power and physicochemical properties, drug loading efficiency, controlled release and longer therapeutic values [75]. They also offer broader-spectrum activity, reduced resistance development and the possibility of targeted delivery over traditional antibiotics [76,77]. These particles exhibit different modes of action such as physical interactions, the disruption of cellular membranes, the induction of oxidative stress and the inhibition of essential cellular processes.

Various types of NPs, e.g., carbon (C-nanotubes, graphene oxide), polymeric (chitosan, poly(lactic-co-glycolic acid), polycaprolactone, polyethylenimine), metallic, lipid-based NPs (liposome, micelles and solid lipid), composite-based NPs (metal–polymer, hybrid lipid–polymer and ceramic) have been developed to offer antimicrobial and anti-biofilm activities against ESKAPE and other pathogens [78,79]. Studies have been conducted on the potent antibacterial capabilities of metal or metal oxide-based NPs such as silver (Ag), copper (Cu) and zinc oxide (ZnO) [80,81,82]. These NPs can interact with bacterial cell membranes, penetrate biofilms and induce oxidative stress, leading to microbial death [83]. Preclinical investigations have shown that ZnO, Cu and Ag NPs target the quorum-sensing system for their antimicrobial activity [78,84]. However, translating NPs into clinical use requires proper synthesis methods and a thorough understanding of their physiochemical properties, pharmacokinetic/pharmacodynamics (PK/PD) and potential toxicity [85,86].

### 3.7. Antimicrobial Peptides (AMPs)

Being naturally occurring short chains of amino acids, typically 15 to 50 residues, antimicrobial peptides (AMPs) are a critical part of the innate immune system of diverse organisms and are ubiquitous in nature [87,88]. They are usually cationic with amphipathic properties, enabling them to interact with microbial membranes and making them potent against a broad spectrum of pathogens [89]. They can neutralise microbial components such as lipopolysaccharides that otherwise mediate immunological responses [90] and act as the first-line defence mechanism against invading pathogens [91]. Their broad-spectrum activity coupled with the rising concern of antimicrobial resistance globally has shifted the focus to AMPs as alternatives to conventional antibiotics [92]. In contrast to conventional antibiotics, bacteria do not easily develop resistance to AMPs because these peptides primarily target their cell membranes, disrupting their integrity and fidelity. This mode of action is more challenging for bacteria to counteract compared to bioprocess-specific targeting processes such as cell wall synthesis or protein translation of conventional antibiotics. Interestingly, many AMPs also exhibit additional modes of action, which intersect with those of conventional antibiotics [93,94]. Meanwhile, several AMPs are able to kill non-replicating bacteria [95].

#### Challenges in the Clinical Translation of AMPs

Despite their significant potential, AMPs face several challenges that hinder their clinical application, such as the high cost of production, susceptibility to proteolysis and toxicity at higher concentrations. Under physiological salt conditions, AMPs may show reduced activity due to impaired electrostatic interactions with membranes. Additionally, they can bind to serum proteins, resulting in diminished efficacy in the presence of serum [96,97,98]. These limitations have restricted the use of AMPs primarily to topical administration [99]. Like other drugs, AMPs undergo a funnel process where only a small percentage advance to clinical trials compared to the total number of compounds initially identified, and an even smaller fraction ultimately achieve marketing approval. Some of the peptide molecules or their mimetics currently undergoing clinical trials for their antimicrobial efficacy include XF-73, Onc72, OP-145, lactoferrin, murepavadin, hLF1-11, C16G2, CLS001 and ramoplanin (NTI-851) [100,101].

The benefits and limitations of these different emerging non-antibiotic approaches against AMR are summarised in Table 1.

## 4. AMP Mimetics and Strategies to Enhance AMP Activity

Different strategies are employed to address the limitations of AMPs to reduce production costs while maintaining their activity, including making AMPs more stable in the presence of proteases, more selective for pathogens and less toxic to host cells, as well as improving bioavailability and half-life in physiological conditions.

Mimetics of AMPs are modifications to or synthetic versions of naturally occurring AMPs that are designed to maintain or improve upon the characteristics of its natural progenitor while improving on its flaws and are called peptidomimetics (which may have modified peptide backbones not based on α-amino acid configuration) [102,103,104].

### 4.1. Peptide Engineering and Chemical Modifications

One of the primary strategies for improving AMP stability and efficacy involves the chemical modification of peptide structures. These modifications aim to enhance resistance to proteolytic degradation, improve membrane selectivity and reduce cytotoxicity.

#### 4.1.1. Peptide Cyclisation

Linear AMPs, with their exposed peptide bonds, are susceptible to chemical and enzyme degradation [105]. The cyclisation of peptides via covalent bonding of N-to C-terminus, side-chain to N- or C-terminus or side-chain to side-chain closes up the peptide, making it rigid and less vulnerable to proteases. There are improvements in selectivity for pathogens, stability and activity in serums and broadening of the spectrum [106,107]. In some cases, however, cyclisation resulted in a loss of activity [108].

#### 4.1.2. Non-Canonical Amino Acid Substitution

Incorporating non-canonical amino acids, including D-amino acids, instead of natural canonical L-amino acids can protect AMPs from degradation by proteolytic enzymes that typically recognise the proteogenic L-amino acids. This modification also maintains antimicrobial activity while enhancing the peptide’s half-life in biological environments and cytotoxicity [109]. D-amino acid substitutions were found to improve the activity of AMP M33 4 to 16 times against Gram-positive bacteria and improved prognosis in MRSA-infected mice [110]. Isomers of α-amino acids, e.g., β- and γ- amino acids, can also be integrated into peptide sequences to reduce the susceptibility to peptide and broaden its spectrum [111,112].

#### 4.1.3. Peptoid-Based Mimetics

As a class of mimetics where side chains are attached to the amino nitrogen atom instead of the α-carbons, peptoids are an interesting alternative to peptides. They offer improved activity and stability [113]. This structural change makes peptoids non-susceptible to proteases [114] while retaining antimicrobial properties. Peptoids have shown strong activity against different microorganisms [115]. Numerous strategies have been employed to develop peptidomimetics, designed to replicate the structure and function of natural melittin peptides while improving their stability [116,117]. In particular, *N*-substituted glycine-based peptoids have emerged as a significant innovation [118]. These peptoids are engineered with alterations in their backbone, making them highly resistant to protease activity while retaining the antimicrobial potency characteristic of antimicrobial peptides (AMPs). *N*-substituted glycine peptoids have demonstrated potent antimicrobial activity against a wide range of pathogens, including bacteria, fungi and viruses including SARS-CoV-2, positioning them as promising broad-spectrum antimicrobials [114,119,120]. Incorporating phenylene or ethylene in molecular structures presents numerous advantages. The rigidity of phenylene and ethylene rings enhances molecular adaptability and electronic properties crucial in electronics and biomedical materials [121]. Their stability and favourable intermolecular interactions contribute to diverse applications offering cost-effective scalability [121]. Studies using X-ray, nuclear magnetic resonance (NMR) and circular dichroism (CD) confirm that the specific orientation of α-helices is essential for peptoid functionality, particularly in biomaterial applications [122]. Notably, peptoids exhibit low immunogenicity akin to AMPs [123].

#### 4.1.4. Lipidation

Lipidation involves attaching fatty-acid chains to AMPs, enhancing their hydrophobicity and ability to integrate into microbial membranes [124]. This modification can significantly boost the antimicrobial activity of peptides by improving their interaction with lipid bilayers, thereby increasing their membrane-disrupting activity [125]. However, excessive hydrophobicity can lead to toxicity towards host cells. Therefore, optimising the balance between hydrophobicity and selectivity is key in designing lipidated AMPs [98].

#### 4.1.5. Conjugation with Functional Groups

AMPs coupled with functional groups, such as polymers, nanoparticles or targeting ligands, can improve their therapeutic properties. Attaching AMPs to polymers such as polyethylene glycol (PEG) can improve their pharmacokinetic profiles [126], increasing circulation time and reducing renal clearance [127]. PEGylation can also reduce the immunogenicity and toxicity of AMPs, making them safer for therapeutic use. AMPs can be encapsulated in or attached to nanoparticles, which protect them from degradation, enhance targeted delivery and allow for controlled release [128,129]. Nanoparticle-based AMP delivery systems have shown promise in overcoming the stability issues that limit the clinical application of free peptides [128].

### 4.2. Use of AMP Combinations

Using combinations of different AMPs, or combining AMPs with traditional antibiotics, can enhance antimicrobial efficacy and reduce the likelihood of resistance development [130]. The synergistic effects between different AMPs can disrupt multiple microbial targets simultaneously, while the use of antibiotics can complement the membrane-disruptive activity of AMPs with intracellular inhibition [131]. For instance, the combination of AMPs with β-lactam antibiotics has been shown to enhance the permeability of bacterial membranes, allowing greater penetration of the antibiotic into the bacterial cell [132]. Some other examples of synergy between antibiotics and AMPs are as follows: (a) LL-37, a 37-aminoacid peptide proteolytically released from the human cathelicidin hCAP-18, and colistin against MDR *E. coli* [133]; (b) Novicidin, a linear cationic α-helical peptide, and rifampin, as well as ceftriaxone and ceftazidime, against MDR *Enterobacteriaceae* [134]; (c) S1-Nal-Nal with vancomycin, ciprofloxacin and tetracycline against *E. faecium* BCRC 15B0132 and *A. baumannii* BCRC 14B0097C [131]. AMPs have also shown synergistic activity with peptoid mimics against *E. coli* [135].

## 5. Mechanisms of Action of AMPs

**Figure 2:**
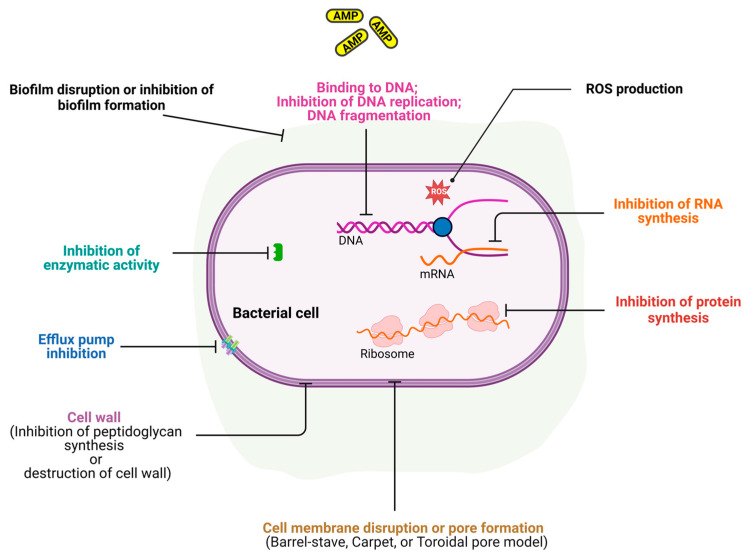
Representation of the mechanisms of action of antimicrobial peptides (AMPs) on bacterial cells (Created in BioRender. Mishra, S. (2024) https://BioRender.com/c41f443, accessed on 25 December 2024).

AMPs possess multiple modes of action against microorganisms. Cationic peptides carry a net positive charge (2 to 9), and in many cases (but not all cases, e.g., Mel4) possess around up to 50% hydrophobic amino acids. Microbial membranes have transmembrane potential values of −130 to −150 mV while mammalian cell membranes have −90 to −110 mV, thus microbial membranes are more electronegative. This feature supports the selective toxicity of AMPs as they are attracted to the negatively charged microbial membranes rather than mammalian membranes [93,136]. For instance, they can bind to Gram-negative bacterial lipopolysaccharide membrane components or lipoteichoic acids in Gram-positive bacterial cells [137]. Additionally, their amphipathic nature enables them to embed themselves in the cell membrane such that their hydrophobic and hydrophilic regions interact with the lipid bilayer [138,139]. This results in several possibilities, as outlined in Figure 2 and the following subsections.

### 5.1. Membrane Disruption

AMPs inserting themselves into the lipid bilayer of target microorganisms can lead to pore formation and the consequent leakage of cytoplasmic content, resulting in death. Poration can occur via mechanisms such as the ‘barrel-stave model’ [140], the ‘carpet model’ [141,142] or the ‘toroidal-pore model’ [143,144]. In the barrel-stave model, aggregates of AMPs in multimeric forms are inserted into the cell membrane’s bilayer and arranged parallel to the phospholipids, forming a channel. The carpet model incorporates the detergent-like destruction of the cell membrane after accumulating on the cell surface. The process of AMPs accumulating and becoming vertically embedded into the cell membrane, followed by bending to form a ring hole, is referred to as the toroidal pore model [145].

### 5.2. Intracellular Targeting

After translocation into the cell, some AMPs act on intracellular components, such as nucleic acids, proteins or enzymes to interrupt essential biological processes [146,147].

### 5.3. Immunomodulatory Effects

Other AMPs promote wound healing, encourage responses from the immune system of the host and recruit immune cells and other relevant immunomodulation, beyond just killing microorganisms [148,149].

## 6. Reduction in Virulence Factors by Peptidomimetics

MDR pathogens express an array of virulence factors, which are cellular components, primarily proteins, that enable bacteria to establish infections. Cell-associated factors, such as structural components, can facilitate adhesion and invasion. Extracellular virulence factors including different toxins and enzymes help in competing for nutrients, acquiring iron and evading host defences. These virulence factors are encoded by specific genes located either on the bacterial chromosome or on mobile genetic elements [150,151]. The presence of antibiotic resistance mechanisms, along with these virulence factors, often results in infections that are difficult to treat, especially in immunocompromised patients. To address the growing trend of antimicrobial resistance, anti-virulence strategies are being explored as an unconventional approach to mitigating MDR infections. These strategies include the use of peptidomimetics targeting bacterial adhesins, secretion systems, biofilms and quorum-sensing systems [152].

## 7. Antifungal Peptides and Their Mimetics

In the fungal kingdom, there are an estimated 2.2 to 3.8 million species based on host association and 11.7 to 13.2 million species using high-throughput sequencing, but only approximately 150,000 species have been described in the literature [153]. The annual incidence of life-threatening invasive fungal infections is estimated to be over 6.55 million cases globally, resulting in approximately 2.55 million deaths directly attributed to fungal infections including invasive aspergillosis, candidemia, pneumocystis pneumonia, cryptococcal meningitis, disseminated histoplasmosis, talaromycosis, mucormycosis, coccidioidomycosis and fungal asthma [154].

To combat these fungal infections, different drug classes exist with different mechanisms, e.g., ergosterol inhibitors/binders (e.g., azoles, polyenes and allylamines), β-1,3-D-glucan synthesis inhibitors (echinocandins) and drugs with intracellular activity (pyrimidine analogues/thymidylate synthase inhibitors and mitotic inhibitors). However, no single antifungal agent is universally suitable for all patients with particular mycosis. Comorbid conditions, hypersensitivities, localised or systemic infection, immune status and the threat posed by infection due to antifungal-resistant strains add further challenges to treatment [155]. The high plasticity of fungal genomes and their ability to evolve, along with their extensive genetic diversity, enables the rapid development of antifungal resistance [156].

**Figure 3:**
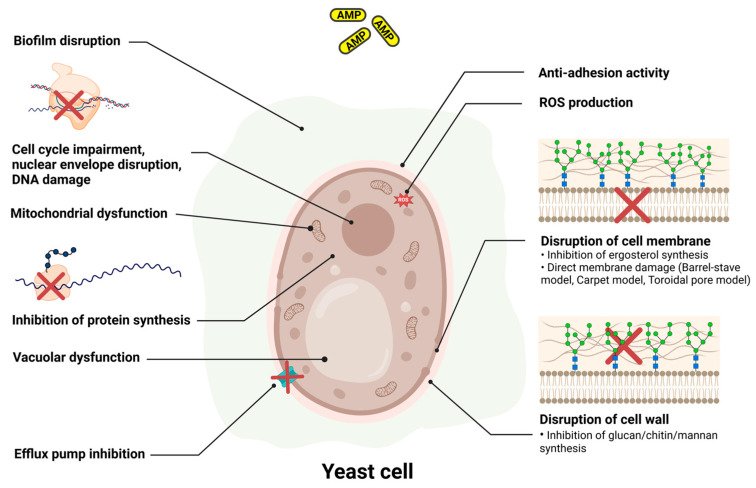
Representation of the mechanism of action of antimicrobial peptides (AMPs) on yeast cells (Created in BioRender. Mishra, S. (2024) https://BioRender.com/p54r433, accessed on 25 December 2024).

There are nearly 1500 antifungal peptides listed in the antimicrobial peptide database (APD) (https://aps.unmc.edu/database/anti, accessed on 1 December 2024) including both natural and synthetic or semisynthetic AMPs. These AMPs have different mechanisms of action against fungi (Figure 3). They can act on and rapidly disrupt the cell membrane, resulting in cell death, or interact with the β-glucan, chitin or mannan in the cell wall. Alternatively, they can target signalling pathways or intracellular components, thereby causing the production of endogenous ROS, the dysfunction of mitochondria and cytoplasmic vacuoles, ATP efflux, the disruption of cation homeostasis and programmed cell death and impairment of the cell cycle [157]. The AMPs that interact with fungal membranes usually possess broad-spectrum activity against bacteria as well, whereas AMPs that target the cell wall are more fungi-specific [158]. Several naturally occurring AMPs can inhibit chitin biosynthesis (nikkomycin Z), destabilise plasma membranes, cause pore formation and damage cell walls (lactoferampic B, lactoferricin B, lactoferricin H, halocidin and magainin-2), lyse spores or perturbate cell walls (cecropin B, osmotin, stomoxyn and temporin B). These, along with several (semi)synthetic AMPs, can inhibit 1,3-β-d-glucan synthase (echinocandins including anidulafungin, caspofungin and micafungin), decrease mitochondrial membrane potential (CGA-N12-0801), induce pore formation or damage cell walls (PPD1, 66-10, C1203TR, PepGAT, *R*cAlb-PepII and Osm-pepA), generate intracellular ROS (*Mo*-CBP_3_-PepI, *Mo*-CBP_3_-PepII, *Mo*-CBP_3_-PepIII and Octominin) and have shown good antifungal activities in vitro [159].

Eechinocandins are lipopeptides that kill *Candida* spp. and are fungistatic against *Aspergillus* spp. These are recommended by the Infectious Diseases Society of America (IDSA) to be used as the first-line therapy against candidemia. However, WHO fungal priority pathogens including *Cryptococcus* spp., *Fusarium* spp., *Scedosporium* and Mucorales are not susceptible to echinocandins [160,161]. Several AMPs are currently in clinical trials including Novexatin^®^ (NP213), Omiganan (MBI-226), PAC113 and CZEN-002, among which NP213 is a fungicidal cationic peptide acting on the fungal outer membrane, effective against onychomycosis [162]. More research is warranted to develop antifungal peptides active against MDR fungi.

Different animal models have been used to evaluate the antifungal properties of AMPs. The efficacy of AMPs in localised or systemic fungal infection models can be assessed by administering peptides through different routes, including mucosal (oral and vaginal), superficial (skin and nails), gastrointestinal and lung or systemic infections (intravenous and intraperitoneal), as applicable [163]. Some of the AMPs that have been tested in vivo as antifungal peptides include HsLin06_18, drosomycin, cathelicidins, EntV, Psoriasin, VL-2397 and sEntV, showing efficacy against pathogenic yeast or moulds [163]. A tripeptoid, AEC5, had significant efficacy and selectivity for *C. neoformans*, achieving a 50% inhibition of fungal growth in half an hour. It also showed an in vivo murine half-life exceeding 20 h and was non-toxic in a mouse model at doses of up to 50 mg/kg over 28 days [164].

AMPs can also provide antifungal coatings to medical devices. For example, the AMP melimine, a cationic hybrid peptide of melittin and protamine, when covalently attached to contact lenses, could reduce the adhesion of *Fusarium solani* ATCC 36031 and *Candida albicans* ATCC 10231 to 1.4 ± 0.2 log_10_ colony-forming units, as well as being able to produce similar reductions in adhesion for drug-resistant *P. aeruginosa* and *S. aureus* and the protozoan *Acanthamoeba castellanii* ATCC 50370 [165].

## 8. Antiviral Peptides and Their Mimetics

**Figure 4:**
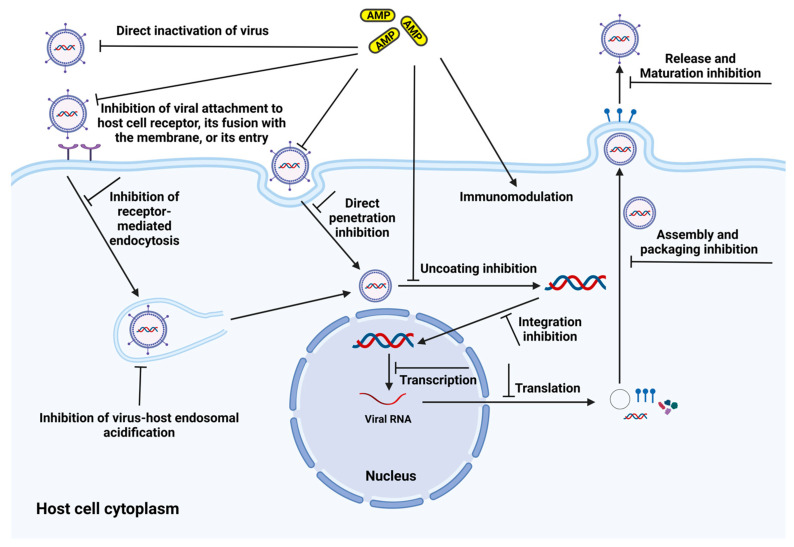
Representation of mechanism of action of antimicrobial peptides (AMPs) on virus. Created in BioRender. Mishra, S. (2025) https://BioRender.com/j49y980, accessed on 25 December 2024.

AMPs have been extensively studied for their antiviral properties and are collectively referred to as antiviral peptides (AVPs) [166]. Over the years, both natural and synthetic peptides have been evaluated for their efficacy against a wide range of viruses, including both enveloped and non-enveloped types [167]. Alongside these peptides, antimicrobial peptidomimetics have also been developed and tested for antiviral activity [168]. Antiviral peptides exhibit diverse structures, ranging from linear to cyclic forms, with cationic to anionic charges and hydrophobic to hydrophilic properties, along with different secondary structures, such as α-helices, β-sheets or random coils. AVPs can target nearly every stage of a virus’s life cycle (Figure 4), with key mechanisms of action including:

(a) Disrupting the viral envelope or membrane: Many AMPs target the viral lipid membrane, destabilising its structure and preventing infection. This is particularly effective against enveloped viruses such as HIV [169], influenza [170], coronaviruses [171,172], hepatitis C [173,174], etc. For non-enveloped viruses like the BK virus, peptides can target the protein membrane, causing virion aggregation [175].
(b) Inhibition of viral entry: Some AMPs block virus attachment and fusion with host cells by targeting virus or cell receptors or coreceptors, thereby preventing the attachment and fusion thereby initiating the infection [176].
(c) Inhibition of viral replication: Several AMPs disrupt viral replication by targeting viral genetic material, interfering with viral proteins or hindering virus assembly during late replication stages [177,178].
(d) Immunomodulation: Certain peptides can stimulate the host’s immune response, preparing cells to combat viral infection more effectively [179,180].

Currently, most peptidomimetics are reported to be effective against enveloped viruses, with limited data available on the efficacy against non-enveloped viruses. AVPs often target the membranes of enveloped viruses [181,182,183,184]. Some peptidomimetics can block HIV entry by targeting specific coreceptors, while others inhibit viral proteins, thereby preventing infection [185]. Although peptidomimetics have opened new avenues for antiviral options, there are still relatively few studies on these compounds. Researchers should focus on understanding the structure–activity relationships of these peptidomimetics to enhance their efficacy against different viruses.

## 9. Antiparasitic Peptides and Their Mimetics

In 2019, an estimated 309 million disability-adjusted life-years (DALYs) were linked to 85 different parasites in children under the age of five, with *Plasmodium* spp., the causative agent of malaria, accounting for 12.0% of the total [186]. Another parasite, *Toxoplasma gondii,* is associated with various infections including congenital toxoplasmosis, which is transmitted from the mother to the foetus. This can range from subclinical infection to spontaneous abortion, and in surviving infants, it may lead to craniocerebral, ocular or cognitive abnormalities and schizophrenia [187]. The evidence of exposure to *T. gondii* is around 30% of the total human population; the seroprevalence is around 90% in some demographic groups [187]. Besides such neglected parasitic zoonoses, *Acanthamoeba* keratitis is another parasitic infection that has been established as the etiological agent of sight-threatening keratitis of approximate 0.5–10% of global microbial keratitis cases, particularly among contact-lens wearers [188]. Pharmaceutical companies are deprioritising many of these parasitic infections to invest in the research of new compounds targeting these agents. As there are no approved vaccines against these pathogens and many medicines for treatment, e.g., anti-toxoplasma medicines, are relatively toxic, the development of novel antimicrobials is warranted.

There are approximately only 350 AMPs exhibiting antiparasitic properties listed in the AMP database (https://aps.unmc.edu/, accessed on 25 December 2024). Testing of the antiparasitic activity of AMPs has focused on hemoflagellates (*Leishmania* spp., *Trypanosoma* spp.) and apicomplexan protozoa (hemoprotozoa, e.g., *Plasmodium* spp. and *Babesia* spp.; intestinal coccidian parasites, e.g., *Cryptosporidium* spp. and *Cyclospora* spp.; and *Toxoplasma* spp.) [189,190]. Some AMPs like melittin, cecropin, cathelicidin, magainin, defensin, temporin, dermaseptin, eumenitin and histatin are active against *Leishmania* spp. [190]. However, there are many amoebae, flagellates, ciliates, coccidia, microsporidia, nematodes, cestodes and trematodes that are clinically relevant but have yet to be tested and need attention in the scientific community.

Our group previously studied the anti-amoebic activity of a peptidomimetic, RK758, against both clinical isolates and type strains of *Acanthamoeba castellanii* and found that its activity is comparable to chlorhexidine, and therefore, it can be developed further as a novel therapeutic agent for the treatment of *Acanthamoeba* keratitis and as an anti-amoebic disinfectant in contact lens solutions [191]. Similarly, polyhomoarginines, arginine-rich peptides, have also been found to be effective against both trophozoites and cysts of *A. castellanii,* further implying the necessity to conduct in vivo *Acanthamoeba* keratitis animal model studies [192].

Though the complex life cycle of parasites presents challenges in antiparasitic peptide development, the development of computational models and tools for predicting peptide activity can help to design compounds effective against different biological stages of their life cycle.

## 10. Overview of Antimicrobial Coatings Using AMPs

AMPs and their mimics are increasingly being used as surface coatings to prevent the transmission of pathogens and reduce biofilm formation. This is particularly relevant in medical settings. When indwelling medical devices become colonised by microbes, especially in the form of biofilms, complete removal of the device often becomes the only viable solution to treat associated infections [193]. Pathogens can persist on inanimate surfaces, posing a risk of transmission. In such instances, antimicrobial surface coatings offer a practical solution, reducing the likelihood of exposure to harmful microorganisms. AMP-functionalised coatings stand out for their rapid and broad-spectrum activity, with low toxicity to host tissues [194]. A variety of chemical strategies, including adsorption, binding, electrospinning and chemical conjugation, can be applied to regulate the attachment and release kinetics of AMPs from surfaces [195].

### 10.1. Antiviral Coatings Using AMPs and Peptidomimetics

Viral pandemics, such as COVID-19, have underscored the importance of antiviral coatings to stop the spread of viruses in public and healthcare environments. Although various antiviral strategies have been tested on materials such as metals and polymers [196], the use of AMPs and mimetics in surface coatings for antiviral purposes is still a relatively new area of investigation. As an example, one study demonstrated the antiviral activity of a peptide-based surface coating against both DNA and RNA viruses [197]. Overall, much remains unknown about the structure–activity relationship between coated peptides and mimetics against viruses, and further research is needed to understand how their immobilised state affects antiviral efficacy compared to their free state in solutions.

### 10.2. Antibacterial Coatings Using AMPs and Peptidomimetics

AMPs and peptidomimetics, when applied as coatings, function by targeting bacterial membranes, entrapping bacteria, forming pores and ultimately causing membrane lysis. Additionally, these coatings can inhibit bacterial biofilm formation [198]. Researchers have improved the stability, biofilm inhibition and antifouling capabilities of peptoids by attaching them to polymer surfaces, creating antimicrobial coatings for medical devices and other applications [199,200]. Research on surface coating with peptides and peptidomimetics has been conducted on a variety of materials, including glass, contact lenses, masks, fabrics, catheters and biomedical implants, demonstrating their effectiveness in preventing bacterial infections [201,202,203].

Glycine-substituted peptoids have proven highly effective in developing antimicrobial coatings for medical devices, including catheters, contact lenses, stents and implants [165,204]. These devices are particularly susceptible to bacterial colonisation, which often leads to biofilm formation and subsequent infections [205,206,207]. The attachment of peptoids to these surfaces has been shown to significantly reduce microbial adhesion and biofilm development [208]. Notably, research indicates that surfaces functionalised with peptoid-based antimicrobials effectively inhibit common pathogens like *S. aureus* and *E. coli*, thereby minimising the risk of device-associated infections [209,210,211].

Various coating strategies have been explored, including a very simple strategy, dopamine-based layers, used to apply AMPs such as melimine, Mel4 and the mimetic RK758 onto glass. These coatings had antibacterial activity against *E. coli*, *P. aeruginosa* and *S. aureus* [212,213]. In another study, the CWR11 peptide was applied to catheters using polydopamine and found to inhibit *E. coli* adhesion [214]. Immobilised cecropin B inhibited both Gram-negative (*P. aeruginosa*) and Gram-positive (*S. aureus*) bacteria when it was coated with dopamine [215].

Other coupling strategies, such as EDC (1-ethyl-3-[3-dimethylaminopropyl] carbodiimide hydrochloride), have been employed to create covalent bonds between AMPs and surfaces. This approach has been used on contact lenses, where melimine and Mel4 successfully inhibited both Gram-positive and Gram-negative bacteria [216]. A similar strategy was applied to coat the same peptides on silicon hydrogel contact lenses [217]. Recently, proteolytically stable antimicrobial peptoids were immobilised on etafilcon contact lenses via different strategies, including EDC/NHS carbodiimide chemistry, oxazoline plasma and plasma ion immersion implantation (PIII). These methods significantly reduced *P. aeruginosa* adherence by over 5 log_10_ colony-forming units per lens [218].

The biocompatibility challenges of AMP-coated devices have been tackled by conjugating therapeutic agents, such as polymers, which serve as immunomodulatory agents to optimise immune responses and boost the effectiveness of AMPs on implanted materials [219]. However, further in vivo studies should be conducted to explore the biocompatibility, durability and sustained activity of such AMP- or peptidomimetic-coated devices against microbes, enabling their advancement towards clinical use [194]. Overall, these studies provide strong evidence supporting the potential use of peptides and their mimetics as surface-coating agents to inhibit bacterial transmission.

## 11. Additional Strategies for Attachment and Functionalisation

To support diverse applications, several other attachment strategies have been explored. Notable methods are outlined below.

### 11.1. Non-Covalent Attachment Strategies

Non-covalent attachment methods utilise physical interactions, such as electrostatic forces, hydrogen bonding or hydrophobic interactions, to bind peptoids to the substrate [220]. Although these methods lack the long-term stability of covalent bonds, they offer the benefit of reversible binding, which is advantageous for applications that require controlled release or temporary antimicrobial activity. This flexibility allows for dynamic coatings and targeted antimicrobial responses [221].

### 11.2. Electrostatics Layer-by-Layer Deposition

The Electrostatic Layer-by-Layer (LbL) assembly technique creates customisable antimicrobial coatings by alternating positively charged peptides and negatively charged polymers [222]. Utilising charged conjugates like laminarin (LAM) and pullulan (PUL) with lysine (K6) and aspartic acid (D6), it forms stable, biocompatible layers on medical devices. When combined with Cu(I)-Catalyzed Azide-Alkyne Cycloaddition (CuAAC), this approach ensures the durable covalent attachment of antimicrobial agents through stable triazole linkages [223]. A similar strategy involves sequentially depositing layers onto negatively charged liposomes composed of dilauroyl phosphatidic acid (DLPA) and dimyristoylphosphatidylcholine (DMPC) [224]. This layered structure enhances stability and functionality, providing precise control over the thickness, charge and permeability—crucial for effective antimicrobial coatings [225]. The resulting linkages anchor AMPs or peptoids securely, offering long-term protection against microbial colonisation and biofilm formation while enabling uniform distribution and controlled composition of antimicrobial agents [226]. Additionally, this method allows precise control over the composition and density of antimicrobial agents, leading to a more effective and evenly distributed antimicrobial layer.

### 11.3. Photo Crosslinking

The combination of light and antimicrobial hydrogels offers a sustainable, precise approach to infection control by enabling targeted and responsive treatments. This approach aligns with sustainable development goals and provides a proactive strategy to combat drug-resistant infections in healthcare settings [227]. Poly(N-alkyl urea peptoids) are synthesised with 1,6-diisocyanohexane, introducing urea groups for hydrogen bonding and chemical modifications. UV photo crosslinking creates stable networks on hydroxyl polymers, enhancing adhesion while imparting antimicrobial and responsive properties for durable coatings [228].

### 11.4. Polymer Brush Grafting

Peptoids can be grafted onto polymer brushes to create dense and stable antimicrobial surfaces, similar to those formed using peptides or polymers [229]. This technique is particularly effective for coating soft or flexible materials like contact lenses and wound dressings [230,231,232]. Unlike peptides, polymer brush grafting with Poly(*N*-substituted glycine) peptoids offers increased flexibility and control over monomer sequences and chain lengths, enabling precise surface design for antifouling properties. This approach allows for the fine-tuning of charge density, hydration and chain length, leading to improved stability and effectiveness in preventing protein adsorption and cell attachment. As a result, peptoid-based brushes serve as a versatile and reliable platform for antifouling applications in both biomedical and industrial settings [229,233].

### 11.5. Silanes

Silanes create stable covalent bonds with substrates, resulting in durable coatings suitable for medical applications [234]. Peptoids, due to their protease resistance, flexible structure and optimised charge distribution, retain their bioactivity when immobilised. Together, silane-coupled peptoid coatings significantly reduce bacterial growth, presenting a promising strategy for preventing biofilm formation on medical devices [235].

### 11.6. Plasma Surfaces

Plasma-treated surfaces can be created by utilising free-radical-generating molecules such as oxygen (O_2_), nitrogen (N_2_), argon (Ar) and methane (CH_4_) [236]. During plasma treatment, ionised gas molecules interact with the surface, producing reactive species like free radicals, ions and electrons [237]. These reactive species can modify the surface chemistry, creating functional groups that enhance adhesion, wettability or reactivity [238]. This technique is particularly effective in improving surface properties for coatings, bonding and the attachment of biomolecules or antimicrobial agents [210].

### 11.7. Chemical Linkers

Click chemistry provides a versatile and efficient approach for attaching peptoids to polymer surfaces [228]. The use of 1,4-butanediol diglycidyl ether and 1-ethyl-3-(3-dimethylaminopropyl) carbodiimide hydrochloride reactions facilitates the formation of stable amide linkages, enhancing the durability and effectiveness of antimicrobial coatings [165,239]. These techniques are widely employed in developing functionalised surfaces for biomedical applications.

### 11.8. Michael Addition

The Michael addition involves reacting thiol groups with activated alkenes, while epoxy-based chemistry uses epoxy groups to form strong covalent bonds with peptoids [240]. Additionally, thiol–ene reactions link peptoids to polymers through the reaction of thiol groups with carbon–carbon double bonds [241]. These methods are particularly effective for coating soft or flexible materials, such as contact lenses and wound dressings, enhancing both antimicrobial properties and stability [242].

## 12. Conclusions

There has been an ongoing arms race between microbes and humans driven by the widespread use of antibiotics and other antimicrobial agents. Bacteria continuously evolve new resistance mechanisms, compelling scientists to develop novel antimicrobial agents with selective toxicity that target these microbes without promoting resistance. AMPs are a promising non-classical antibiotic or antimicrobial class with several advantages for combating antimicrobial resistance. However, their clinical potential is limited by several challenges, including their instability in clinically relevant environments, susceptibility to protease degradation, toxicity to host cells and high development and production costs. Strategies such as lipidation, modifying peptides into peptidomimetics and combining AMPs with other peptides or conventional antibiotics offer opportunities to enhance their stability, safety and efficacy making them more suitable for clinical applications. Therefore, the quest for new alternatives to treat infections should focus not only on AMPs but also on their combination with other active molecules.

AMPs and their synthetic mimetics represent a new frontier in antimicrobial coatings, offering broad-spectrum protection against pathogens. Their ability to disrupt microbial membranes and prevent pathogen adhesion makes them an invaluable tool in the fight against infectious diseases. As research continues to optimise the stability, efficacy and cost-effectiveness of AMP-based coatings, these materials will likely become integral components in healthcare, public safety and everyday consumer products, helping to prevent the spread of pathogens in a post-pandemic world. Furthermore, due to a limited number of in vivo studies focusing on the efficacy of antimicrobial peptidomimetics on microbes, their advancement into clinical trials and practical healthcare applications is significantly hampered. Therefore, further research should focus on in vivo studies to evaluate both the antimicrobial and anti-virulence efficacy of these promising molecules.

## Figures and Tables

**Table 1:** Summary of pros and cons of different non-antibiotic approaches.

Antimicrobial Approaches	Advantages	Limitations
Bacteriophages	Highly specific to infecting bacteria, minimising off-target effects Only low dosages are required for treatment Bactericidal effect Capable of evolving alongside the target microbe, potentially reducing resistance development Suitable for topical applications, providing localised treatment options Low toxicity and good stability at low temperatures and inside human body Independent of antibiogram of targeted bacteria Reversion of antibiotic resistance when bacteriophage resistance develops Phage cocktails minimise the chances of resistance emergence Occur naturally Self-replicating, self-dosing, self-limiting	Not suitable as empirical treatment because of narrow-spectrum activity Limited ability to penetrate and act on intracellular bacteria Delivery challenges due to rapid clearance by the immune system Often require adjunctive use of antibiotics or other therapies. High cost for personalised phage therapy and maintaining phage banks Regulatory pathways for approval remain unclear and complex Risk of bacteria developing resistance to phages Both clinical trials and pharmacokinetic/pharmacodynamic (PK/PD) studies are limited Standardised phage susceptibility testing reference method is lacking As phages are naturally occurring, there may be an established immune system response to them, and there can also be patent issues, and commercial corporations may not invest in phage therapy research Symbiotic as well as predatory relationships with biofilms Can transfer toxin genes between bacteria
Probiotics	Natural, biodegradable, generally regarded as safe for continuous use with no side effects or minimal toxicity Promotes the growth of beneficial microbes without disrupting the natural microbiome Boost host immune responses and support overall gut health Reduce infection risk by competing with pathogens for nutrients and adhesion sites Helpful to reduce selective pressure for resistant bacteria. Cost-effective Easy to apply	Benefits may be temporary, requiring long-term treatment Effects are often general, and may not specifically target pathogens Strain-specific benefits can vary greatly between individuals and conditions Though rare, they can cause invasive infections, particularly in immunocompromised hosts Lack of standardised regulatory frameworks for probiotic products Action is slower than antibiotics in managing infections Sensitivity under extreme stress conditions, e.g., acidity, moisture and temperature Transfer of antibiotic resistance genes of the probiotic bacteria to intestinal microbes can result in antimicrobial resistance Deleterious metabolic activities
Immunotherapies	Rapid action High specificity Minimal disruption to host normal microbiota Some immunotherapies can provide long-term protection by training the immune system	Can be unstable High cost Require precise control of the molecular size, shape, affinity and valency Constantly mutating targets can affect activity Risk of immunogenicity Possibility of late-onset toxicity
Photodynamic therapies	Broad-spectrum activity Can be highly specific Irradiation confined to the infected site, minimising systemic toxicity Repeated treatments do not lead to development of resistance Multiple bacterial targets Enable tailored and personalised treatment	Active only during light exposure which leads to surviving microbes regrowing and sustaining infection Limited to local infections as it requires light activation of photosensitisers Must be administered by healthcare professionals in clinical settings Time consuming Bacterial efflux pumps reduce the effectiveness of photodynamic therapies using methylene blue by lowering its intracellular accumulation and the corresponding production of intracellular reactive oxygen species (ROS)
Essential oils	Derived from natural products making them an eco-friendly option Broad-spectrum activity Reduced likelihood of microbial resistance Offer multiple modes of antimicrobial activity Can reverse bacterial resistance to antibiotics	Low solubility (lipophilicity) Can be harmful via ingestion or dermal exposure; potential side effects include headache, bleeding, eye irritation, asthma, dermatitis, neurotoxicity, genotoxicity and immunotoxicity Can cause host cell membrane damage Easy degradation, high volatility and photosensitivity Variability in composition complicates standardisation and application in industry Limited legislative frameworks for therapeutic and industrial applications
Nanoparticles	Multiple modes of antimicrobial activity Broad-spectrum of activity Targeted drug delivery via accumulation Fewer side effects Can cross blood–brain barrier Good therapeutic index Controlled release of drugs resulting in extended therapeutic lifetime Can be used in synergistic combination with antibiotics Can enhance antimicrobial activities through photothermal therapy and photodynamic therapy Possess antibiofilm activity as well	Difficult to ensure surface stability and accessibility Require optimisation of doses which is problematic Demand identification of appropriate administration routes Can enter host cells and cause oxidative stress, DNA damage, inflammation and other toxic events Nanotoxicity Lack of characterisation methods unaffected by NPs properties Prolonged and widespread use can result in microbial resistance Impact on ecosystems with the release of antimicrobial NPs into the environment Beneficial normal microbiota can also be affected in body Demands stringent regulatory protocols for successful applications Antimicrobial properties of NPs or implant surfaces can be affected by protein contamination
Antimicrobial peptides	Broad-spectrum of activity Multiple modes of action High bactericidal activity Active even at low concentration Good water solubility Rapid action Low risk of development of resistance in microbes Synergise with antibiotics Can be combined with nanoparticles Less stability in the environment ensures less chances of development of antimicrobial resistance in environmental microbes Active against both planktonic and biofilm cells Can be modified into peptidomimetics, which provides protease resistance, metabolic stability and retained antimicrobial activity AMPs with short sequences are easier to synthesise Low synthetic cost as peptidomimetics	Short half-life due to susceptibility to proteases, enhanced hepatic and renal clearance Susceptibility to physiological salt concentrations Potential cytotoxicity or haemolytic activity Poor penetration of intestinal mucosa Can exhibit immunogenicity Some bacteria are intrinsically resistant to AMPs

## Data Availability

Not applicable.
